# Assessment of hemodynamic responses to exercise in aortic coarctation using MRI-ergometry in combination with computational fluid dynamics

**DOI:** 10.1038/s41598-020-75689-z

**Published:** 2020-11-03

**Authors:** Charlotte Schubert, Jan Brüning, Leonid Goubergrits, Anja Hennemuth, Felix Berger, Titus Kühne, Marcus Kelm

**Affiliations:** 1 Institute for Imaging Science and Computational Modelling in Cardiovascular Medicine, Charité-Universitätsmedizin Berlin, Berlin, Germany; 2 Department of Congenital Heart Disease, Deutsches Herzzentrum Berlin (DHZB), Berlin, Germany; 3 DZHK (German Centre for Cardiovascular Research), Partner Site Berlin, Berlin, Germany; 4 Einstein Center Digital Future, Berlin, Germany; 5 Berlin Institute of Health (BIH), Berlin, Germany

**Keywords:** Congenital heart defects, Aortic diseases, Magnetic resonance imaging

## Abstract

In patients with aortic coarctation it would be desirable to assess pressure gradients as well as information about blood flow profiles at rest and during exercise. We aimed to assess the hemodynamic responses to physical exercise by combining MRI-ergometry with computational fluid dynamics (CFD). MRI was performed on 20 patients with aortic coarctation (13 men, 7 women, mean age 21.5 ± 13.7 years) at rest and during ergometry. Peak systolic pressure gradients, wall shear stress (WSS), secondary flow degree (SFD) and normalized flow displacement (NFD) were calculated using CFD. Stroke volume was determined based on MRI. On average, the pressure gradient was 18.0 ± 16.6 mmHg at rest and increased to 28.5 ± 22.6 mmHg (p < 0.001) during exercise. A significant increase in cardiac index was observed (p < 0.001), which was mainly driven by an increase in heart rate (p < 0.001). WSS significantly increased during exercise (p = 0.006), whereas SFD and NFD remained unchanged. The combination of MRI-ergometry with CFD allows assessing pressure gradients as well as flow profiles during physical exercise. This concept has the potential to serve as an alternative to cardiac catheterization with pharmacological stress testing and provides hemodynamic information valuable for studying the pathophysiology of aortic coarctation.

## Introduction

Aortic coarctation, a congenital narrowing of the aorta, may in the long-term lead to several complications such as arterial hypertension^1^, left ventricular dysfunction^2^ or aortic and intracranial aneurysm formation^3^. Their underlying mechanisms and relevance of different hemodynamic factors in the pathophysiology is a matter of ongoing debate.

In the current clinical decision-making process pressure gradients across the aortic narrowing are one of the decisive factors. As pressure gradients increase during exercise due to increased cardiac output^4,5^, patients with gradients below the threshold for intervention at rest may develop pathologically high gradients during exercise. Therefore, in the case of a borderline indication for surgical or catheter-based treatment, stress tests can help to unmask the hemodynamic relevance of the stenosis.

Commonly used non-invasive methods for determining pressure gradients at rest or during physical exercise (e.g. echocardiography, cuff measurements) are often inaccurate^6–8^. Furthermore, the diagnostic value of exercise blood pressure remains unclear as it has been shown that peak systolic blood pressure does not correlate with the repair site gradient or ventricular mass and does not differ from healthy controls^9^. Alternatively, adrenergic drug infusion can be used during cardiac catheterization, simulating physical exercise, while measuring peak-to-peak gradients^5^. However, the hemodynamic response during pharmacological stress is far from representing responses to physical exercise^10,11^.

The combination of MRI-ergometry and computational fluid dynamics (CFD) could be an approach to quantitatively assess the hemodynamic response to physical exercise. MRI-based CFD for assessing pressure gradients was repeatedly validated against invasive heart catheterization as a reference standard^12–15^. The feasibility of MRI-ergometry has been tested in several previous studies^16–18^. Combining these two methods, would allow an accurate and non-invasive assessment of pressure gradients, aortic and left ventricular hemodynamics at rest and during physical exercise, without the need for adrenergic drugs to simulate exercise. To the best of our knowledge, no such combined approach was reported yet. Apart from clinical implications, this is a promising approach to study the complex pathophysiology of aortic coarctation and how it changes during stress conditions^2^.

Therefore, we aimed to demonstrate the feasibility of non-invasive estimation of transstenotic pressure gradients, aortic flow patterns and left ventricular stroke volume at rest and during physical exercise using an MRI-compatible step ergometer in combination with a previously validated method for numerical simulation of peak-systolic hemodynamics in aortic coarctations. Using this approach, the patient-specific increase in transstenotic pressure-gradient that results from the individual hemodynamic response to dynamic exercise as well as the individual anatomical configuration of the stenosis was estimated.

## Materials and methods

### Study population and design

In this prospective study, patients with aortic coarctation, who underwent cardiac MRI due to elevated Doppler gradients or follow-up after an intervention, were assessed for eligibility between November 2018 and September 2019. Exclusion criteria were the inability to perform ergometry, complex anatomical malformation and general contraindications to MRI. The study protocol was in agreement with the principles outlined in the Declaration of Helsinki and was approved by the Medical Ethics Review Committee (Ethikkomission Charité-Universtätsmedizin Berlin). Written informed consent was obtained from all patients or their guardians.

**Figure 1:**
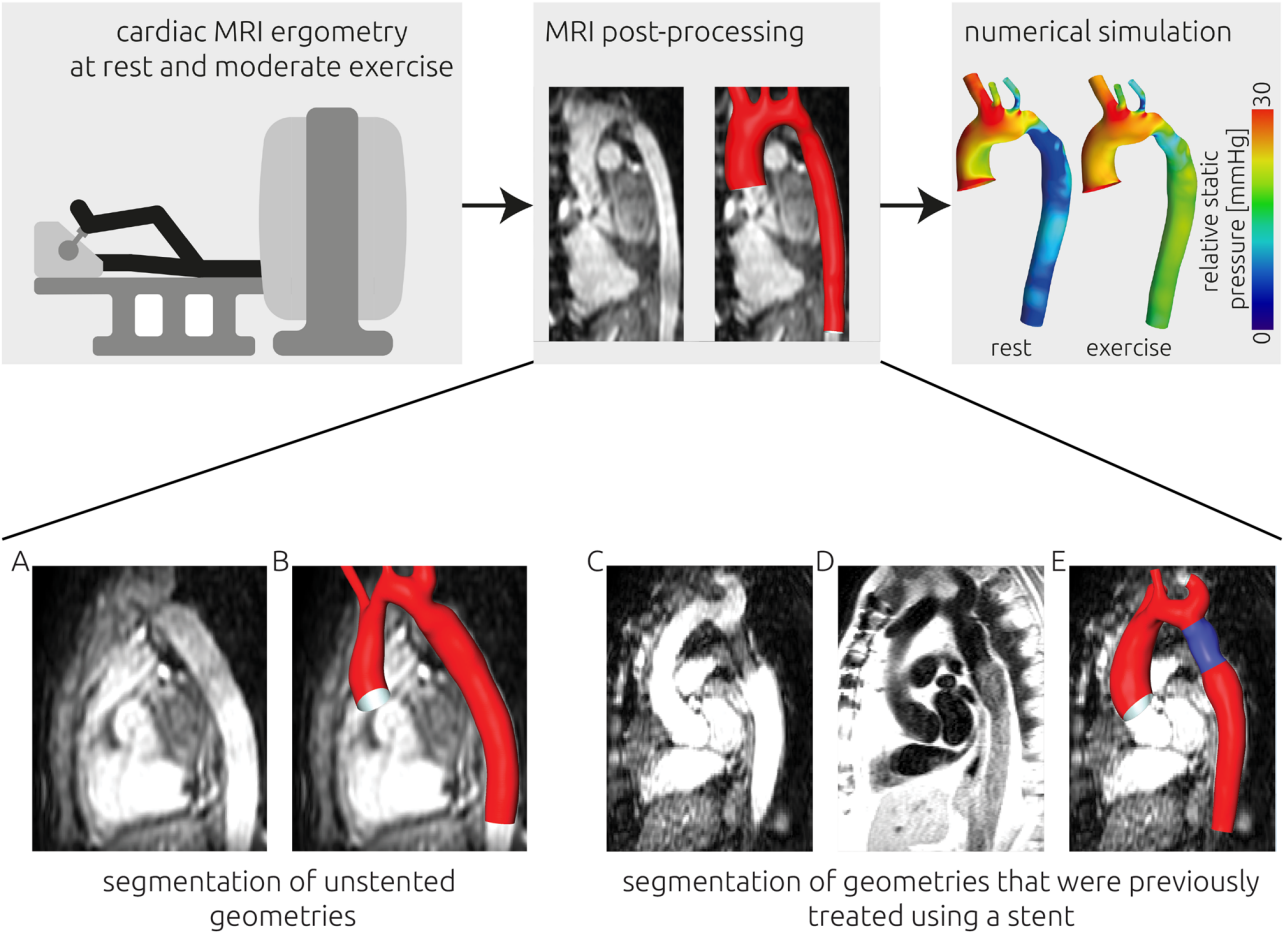
(Upper panel) Visual illustration of the study design. Cardiac MRI of 20 participants with aortic coarctation was acquired during rest and moderate exercise. An absolute increase in heart rate of 50 bpm was targeted during exercise. Using the MRI images, the participant-specific aortic geometry was reconstructed. Using computational fluid dynamics, the transstenotic pressure gradient was calculated during rest and exercise. (Lower panel) Visualization of the image data used for segmentation. The participant-specific anatomy of the aorta was segmented from 3D SSFP (steady-state free precession) images (**A**,**C**). If the respective participant was treated using a stent, additional image information from black blood MR sequences (**D**) was used to improve the segmentation of the stented region of the aorta. An example from only 3D SSFP images is shown in panel (**B**), whereas a combined segmentation of a previously stented patient is shown in panel (**E**).

The study´s workflow is illustrated in Fig. 1. The primary outcome measure was the pressure gradient across the stenosis at rest and during exercise. Secondary outcomes were wall shear stress (WSS), secondary flow degree (SFD), normalized flow displacement (NFD), cardiac index, stroke volume index and heart rate, at rest and during exercise.

### Image acquisition

Data were acquired on a 1.5 T clinical MR system (Achieva; Philips Healthcare, Best, Netherlands) with a five-element cardiac phased-array coil. The cardiac MRI protocol included:

1. Balanced 3D steady-state free -precession (SSFP) imaging of the thoracic aorta (voxel size acquired/reconstructed 2 × 2 × 4/1 × 1 × 2 mm^3^, TE 4 ms, TR 2 ms, flip angle 90°, 3 signal averages, navigator gated, ECG triggered).
2. Black blood turbo spin-echo imaging was performed in patients with metal artifacts (voxel size acquired/reconstructed 1.5 × 1.5 × 6/1.5 × 1.5 × 6 mm^3^, TE 1.4 ms, TR 27 ms, flip angle 90°, prospective triggering in end-diastole).
3. Velocity-encoded MRI (4D VEC MRI) was acquired in planes perpendicular to the ascending aorta distally to the valve and in the descending aorta at the level of the diaphragm to assess inflow conditions in 3 flow encoding orientations and outflow towards the abdominal aorta (voxel size acquired/reconstructed 2 × 2 × 7/1 × 1 × 3 mm^3^, TE 4 ms, TR 3 ms, flip angle 15°, 30 automatically reconstructed phases, retrospective cardiac gating). Note, that 4D refers to three velocity components and the temporal resolution, no volumetric measurement was performed. Velocity encoding was adapted according to the degree of stenosis and was between 3 m/s and 4 m/s. 2D VEC MRI sequences were run at the same position as well as in a plane perpendicular to the descending aorta (voxel size acquired/reconstructed 2 × 2 × 5/1 × 1 × 5 mm^3^, TE 4 ms, TR 3 ms, flip angle 15°). Velocity-encoded MRI was repeated under exercise conditions.

Exercise was performed on an MRI-compatible step ergometer (Ergospect Cardio-Stepper, Ergospect, Innsbruck, Austria). To enhance the quality of images, the aim of exercise performance was to maintain a moderate exercise level without exertion, which was defined as 50 beats per minute above the resting heart rate^19^. All patients underwent blood pressure measurements of the upper and lower extremities (Dinamap Pro 300V2, GE Medical Systems, Fairfield, CT, USA) at rest and blood pressure measurements of the upper extremeties as well as heart rate monitoring at rest and during exercise.

### Post-processing

All images were processed using ViewForum (R6.3V1L7 SP1, 2010, Philips Medical Systems, Best, Netherlands). The 2D VEC MRI sequences at the level of the ascending aorta were used to assess aortic stroke volume, from which the cardiac index was calculated (heart rate x stroke volume/ body surface area). Here, the respective vessel circumference was manually traced to specify a region of interest. For this region of interest, volume flow curves were generated. Those flow curves were then used to calculate the aforementioned hemodynamic parameters. MRI analysis was performed by M.K., who has a level 3 qualification of the Society of Cardiovascular Magnetic Resonance since 2014. A detailed description of this process is provided in earlier studies^20,21^.

The anatomy of the left ventricle and aorta were segmented based on the diastolic 3D SSFP cine images, using ZIBAmira (v. 2015.28, Zuse Institute Berlin, Germany), following an approach described earlier^22^. Briefly, image voxels belonging to the structures of interest were labelled using semi-automatic tools as thresholding and flood-filling algorithms as well as extensive manual interaction. Within stented regions with poor 3D SSFP signal quality, black blood sequences were used to reconstruct the stent configuration using the same tools as described earlier. Due to minor movements by the patients, registration of black blood and 3D SSFP segmentation was necessary in some cases. This was performed using the affine registration tool provided by ZIBAmira. Subsequently, both segmentations were merged to obtain a complete segmentation of the patient-specific aorta. A smooth surface geometry was generated using a volume-preserving algorithm^23^. The ascending aorta was cut using the 4D VEC MRI measurement´s plane (sinotubular junction). The process is illustrated in Fig. 1.

### Computational fluid dynamics (CFD) simulation

Numerical simulations for calculation of patient-specific hemodynamics at rest and during exercise were performed using an approach that was previously validated against in-vivo catheter-based measurements^12^ as well as 4D-flow-MRI measurements^15^ using STAR-CCM + (v. 14.02, Siemens PLM Software, Plano, TX 75024, USA). A finite volume representation with a cell size of 0.4 mm was generated using the polyhedral meshing algorithm provided by STAR-CCM + . To adequately resolve the flow adjacent to the wall a boundary layer consisting of 3 layers was added. The overall thickness of the boundary layer was one third of the average cell size at the wall and each layer’s thickness was 50 percent larger than that of the previous layer. The meshing algorithm also was able to reduce the local mesh size down to 0.1 mm in regions with high curvatures. This mesh resolution was shown to yield mesh independent results in earlier studies^22^.

A no-slip boundary condition was assumed for the aortic wall and the aorta was modelled as rigid. At the descending aorta, the maximal volume flow rate measured using the 4D QF sequence during rest and exercise was applied as outlet boundary condition. At the ascending aorta, the patient-specific, peak systolic velocity vector profiles, which were measured using planar 4D VEC MRI at rest and during exercise, were applied. Therefore, while the same segmentation was used for simulation of hemodynamics at rest and during exercise, the inlet and outlet boundary conditions were modelled using the 4D-flow-MRI measurements of the respective condition. The velocity information was first exported using GTFlow (v. 3.2.3, gyrotools, Switzerland). Using MATLAB (v. 2018a, The Mathworks Inc., USA) this velocity information was mapped onto the meshed inlet using a linear interpolation scheme. In four cases, the 2D VEC MRI had to be used due to misaligned planes or aliasing in the 4D data. The volume flow difference between ascending and descending aorta was specified at the vessels branching from the aortic arch using two assumptions: The volume flow was equally distributed within the brachiocephalic artery (right arm and head) as well as the left common carotid artery and left subclavian artery. At subsequent bifurcations, the volume flow distribution was specified using Murray’s Law^24^.

Blood was modelled as non-Newtonian fluid using a Carreau–Yasuda model described by Karimi et al.^25^ and with parameters provided by Abraham et al.^26^. A two-equation k-ω-SST was used to model the expected turbulence in the aorta. A turbulence intensity of 5 percent was assumed at the ascending aorta.

**Figure 2:**
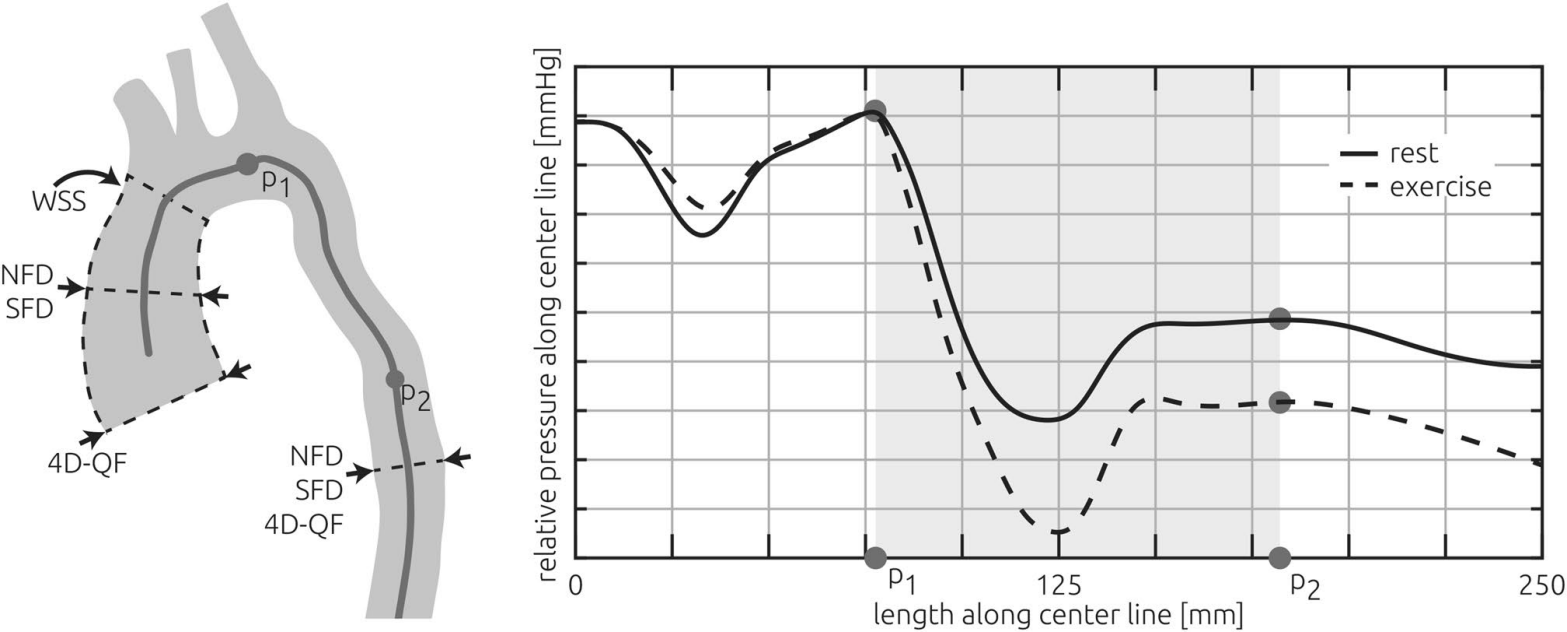
Illustration of all measurements and calculations reported in this study. The patient-specific volume flow rate was measured using 4D VEC MRI (4D velocity-encoded MRI, abbreviated 4D-QF) at the sinotubular junction as well as the descending aorta. Those measurements as well as the patient-specific anatomy (grey vessel shape) were used as boundary conditions for the numerical simulations which were used to calculate all other parameters. Secondary flow degree (SFD) and normalized flow displacement (NFD) were calculated at the mid-section of the ascending aorta and the descending aorta. The surface-average of calculated wall shear stresses (WSS) was evaluated for the ascending aorta. The pressure gradient was calculated using the pressure upstream (P1) and downstream of the stenosis (P2), where the pressure recovery due to deceleration of the blood flow was maximal (right panel).

The following parameters were evaluated using the hemodynamic simulations at rest and during exercise: The pressure gradient across the coarctation; surface-averaged WSS in the ascending aorta; SFD, which is the ratio between the mean in-plane velocity magnitude and the mean through-plane velocity; and the NFD, describing the position of the velocity maximum within the vessel in relation to the vessel radius^27^. The latter two parameters were evaluated at the level of the sinotubular junction as well as the ascending aorta and descending aorta (Fig. 2). Segmentations and numerical simulations were performed by J.B., with 11 years of experience.

### Statistical analysis

Statistical analysis was performed using IBM SPSS Statistics 25 (IBM Inc., USA). Data distribution was examined using the Shapiro–Wilk test. Differences between rest and exercise were compared using paired t-tests for normally distributed data and Wilcoxon signed-rank test for non-normally distributed data. A p-value < 0.05 was considered significant.

## Results

Twenty-four patients were assessed for eligibility. Three patients were excluded due to significant malformation (e.g. extra anatomical bypass and Fontan circulation). In one patient a trigger error occurred during VEC MRI acquisition. The final analysis was carried out in the remaining 20 patients. The baseline characteristics of the included patients are shown in Table 1. Of the 20 patients (13 men, 7 women) included, 3 patients were untreated, 5 had undergone surgical repair by end-to-end-anastomosis, 11 were treated by stent-implantation and one stented patient was also treated surgically. The mean age of included patients was 21.5 ± 13.7 years. No symptoms occurred during exercise. The average workload present during exercise was 83.5 ± 37.8 W. On average, 85.79 ± 10.28% of the calculated target heart rate was reached during exercise. Patients’ systolic blood pressure, measured using cuff-measurements at the arm, significantly increased from rest to exercise (128.45 ± 21.45 to 158.65 ± 33.97 mmHg, p = 0.002).

**Table 1:** Baseline characteristics of included patients.

Parameter	Patients (n = 20)
Men	13 (65%)
Age (years)	21.5 ± 13.7
Weight (kg)	59.4 ± 20.4
Height (cm)	163.8 ± 16.4
BSA (m^2^)	1.6 ± 0.4
Degree of stenosis (%)	35.1 ± 18.1
**Prior interventions**	
Stent	11 (55%)
End-to-End Anastomosis	5 (25%)
Both	1 (5%)
None (native coarctation)	3 (15%)
**Medication**	
Beta blocker	5 (25%)
Diuretics	1 (5%)
ARB or ACE-I	8 (40%)
Calcium antagonists	3 (15%)
**Hypertension**	
Normal	4 (20%)
Elevated	4 (20%)
Stage 1	6 (30%)
Stage 2	5 (25%)
Hypertensive crisis	1 (5%)

Values are mean ± SD or numbers (%).

*BSA* body surface area, *ARB* angiotensin receptor blocker, *ACE-I* angiotensin-converting enzyme-inhibitor.

**Figure 3:**
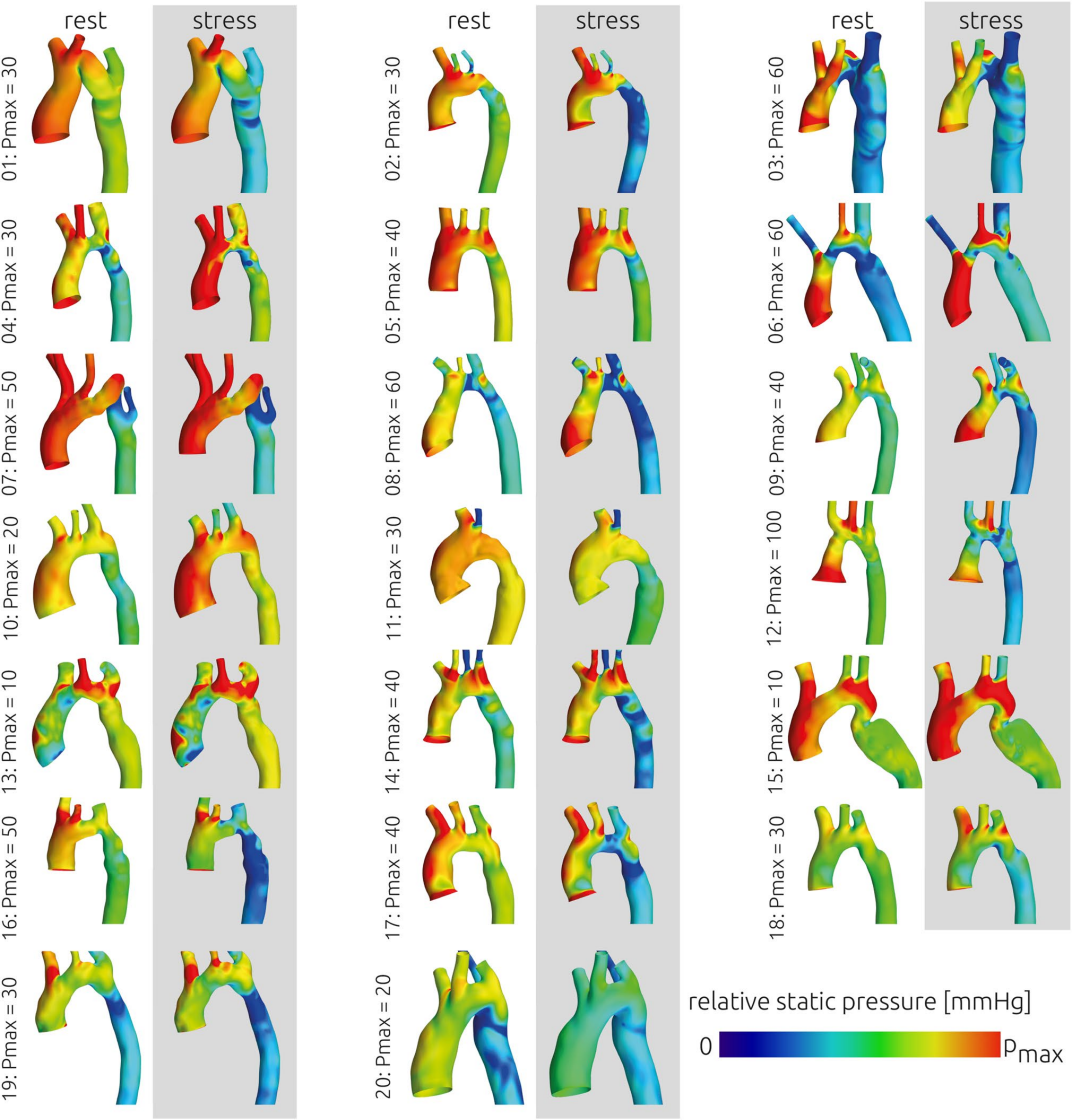
Overview of the relative static pressure distribution that was calculated for all patients at rest and during exercise using computational fluid dynamics. As the individual pressure gradients varied, the upper scale of the colour bar (p_max_) is given next to each patient.

**Figure 4:**
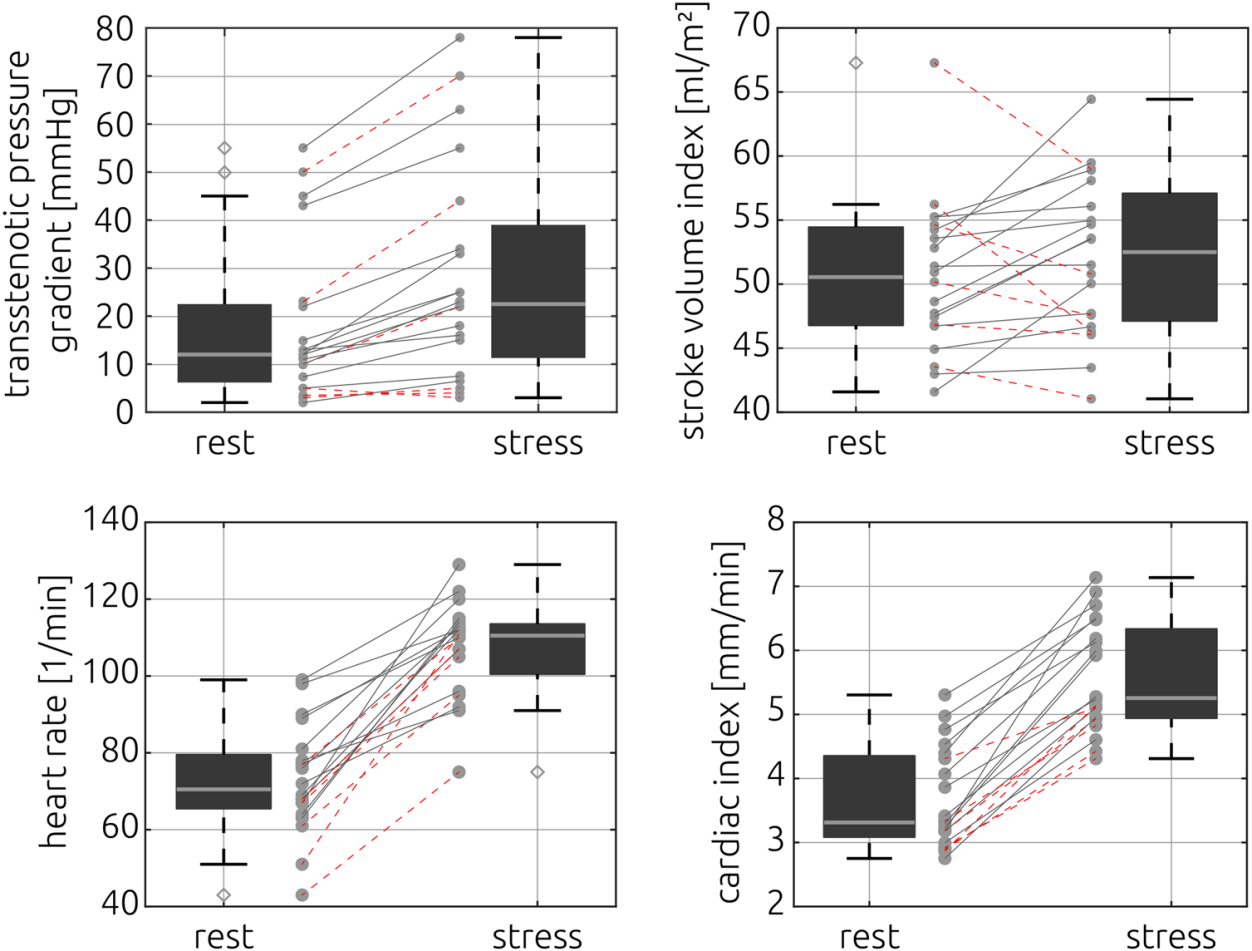
Transstenotic pressure gradients (upper left), left ventricular stroke volume index (upper right), heart rate (lower left) and cardiac index (lower right) at rest and during exercise. Individual cases where the stroke volume decreased during exercise are highlighted using stroked, red lines in all panels.

The relative static pressure distributions at rest and during exercise that were calculated using CFD are shown in Fig. 3 for all patients. The mean transstenotic pressure gradient was 17.99 ± 16.61 mmHg at rest and 28.45 ± 22.56 mmHg during stress (Fig. 4). The pressure gradient significantly increased (p < 0.001) by 73.53%. No correlation between the increase in systolic blood pressure measured at the arm using a cuff and the increase in calculated pressure gradients was observed (|r|< 0.1). In six patients calculated pressure gradients were above the threshold for intervention of 20 mmHg at rest and during exercise. In eight patients pressure gradients remained below 20 mmHg at rest and during exercise. In the remaining six patients calculated pressure gradients were at rest below the threshold for intervention and increased during exercise above 20 mmHg.

Calculated averages and standard deviations for WSS as well as SFD and NFD of the ascending and descending aorta are specified in Table 2. No significant changes in either SFD or NFD were found (SFD ascending aorta, p = 0.14, descending aorta p = 0.53, NFD ascending aorta p = 0.86, descending aorta p = 0.53). However, a significant increase of the surface-averaged WSS in the ascending aorta was observed during exercise (p = 0.006).

**Table 2:** Measured and calculated parameters.

Parameter	Rest (n = 20)	Exercise (n = 20)	P value
Watt		83.5 ± 37.8	< 0.001
Right arm blood pressure systolic (mmHg)	128.5 ± 21.5	158.7 ± 34	0.002
Right arm blood pressure diastolic (mmHg)	64.8 ± 9.1	78.8 ± 19.2	0.01
Heart rate (bpm)	72.9 ± 14.2	107.4 ± 12.4	< 0.001
Stroke volume index (ml/m^2^)	50.6 ± 6	52.2 ± 6.1	0.28
Volume flow rate ascending aorta (ml/s)	407.0 ± 87.3	494.4 ± 146.5	< 0.001
Volume flow rate descending aorta (ml/s)	225.5 ± 76.7	274.8 ± 83.6	0.002
Cardiac index (l/min/m^2^)	3.7 ± 0.8	5.6 ± 0.9	< 0.001
Pressure gradient (mmHg)	17.99 ± 16.61	28.45 ± 22.56	< 0.001
SFD ascending aorta	0.46 ± 0.39	0.42 ± 0.31	0.14
SFD descending aorta	0.5 ± 0.45	0.58 ± 0.66	0.53
NFD ascending aorta	0.08 ± 0.06	0.08 ± 0.06	0.86
NFD descending aorta	0.11 ± 0.06	0.12 ± 0.06	0.53
WSS (Pa)	20.33 ± 12.20	25.37 ± 14.84	0.01

Values are mean ± SD or numbers (%). Differences between rest and exercise were compared using paired t-tests for normally distributed data and Wilcoxon signed-rank test for non-normally distributed data.

*SFD* secondary flow degree, *NFD* normalized flow displacement, *WSS* wall shear stress.

Cardiac index significantly increased by 55.4% from 3.67 ± 0.79 to 5.59 ± 0.86 l/min/m^2^, p < 0.001 and heart rate significantly increased by 46.16% from 72.9 ± 14.19 to 107.4 ± 12.37 bpm, p < 0.001 (Fig. 4). Neither the stroke volume (rest 81.9 ± 19.3 stress 84.1 ± 18.0 ml/min/m^2^, p = 0.28) nor the stroke volume index (rest 50.62 ± 5.97 stress 52.20 ± 6.11 ml/min/m^2^, p = 0.28, Fig. 4) did increase from rest to stress. Individual information regarding general patient information and parameters measured using MRI are given in Table 3 whereas all parameters calculated using CFD are given in Table 4.

**Table 3:** Individual values of general patient information, cuff blood pressure measurements and parameters measured using MRI.

General patient information				Cuff-based blood pressure measurements at the right arm [mmHg]				Data measured using MRI							
ID	BSA [m^2^]	Sex	Age [years]					Heart rate [1/min]		Stroke volume [ml]		Peak-systolic volume flow rate [ml/s]			
				Systolic		Diastolic						Ascending aorta		Descending aorta	
				Rest	Exercise	Rest	Exercise	Rest	Exercise	Rest	Exercise	Rest	Exercise	Rest	Exercise
1	1.51	F	32	126	187	65	125	50	101	84.9	69.9	338	411	231	352
2	1.87	M	21	139	149	67	67	83	129	84.0	87.3	459	581	305	433
3	1.78	F	12	102	114	58	55	85	132	89.3	84.7	456	466	229	269
4	1.87	M	18	150	163	76	72	85	150	88.7	100.2	518	782	301	317
5	1.83	M	21	136	172	63	90	85	119	93.2	106.3	551	645	292	378
6	1.51	M	13	145	149	70	69	94	125	80.9	83.0	445	533	199	210
7	1.07	M	8	133	160	66	71	83	115	55.0	55.1	243	250	128	154
8	1.98	M	15	129	142	58	61	44	51	133.2	116.8	464	635	294	384
9	1.06	M	10	129	134	57	71	68	99	56.0	68.3	303	440	117	164
10	1.65	F	33	120	168	68	82	66	74	77.3	76.0	341	411	226	175
11	1.64	F	27	123	138	67	86	57	87	89.6	83.3	423	470	176	260
12	1.53	F	14	115	169	54	70	65	95	71.5	73.0	318	443	188	253
13	2.20	M	51	120	134	71	67	65	93	95.8	90.3	493	526	226	258
14	1.32	M	14	87	204	52	98	79	89	71.6	78.5	434	431	220	284
15	2.14	M	59	191	185	93	85	69	76	89.0	107.1	501	632	218	264
16	1.69	M	15	136	264	61	128	99	62	82.2	92.4	453	603	173	229
17	1.54	M	13	120	126	56	71	76	86	73.5	82.4	411	584	204	340
18	0.82	M	8	126	138	69	73	94	112	45.3	48.3	269	275	86	123
19	1.96	F	17	142	123	65	66	85	96	108.3	109.9	408	582	270	347
20	1.61	F	29	100	154	60	69	67	103	69.2	70.0	312	426	189	304

**Table 4:** Individual values of parameters calculated using computational fluid dynamics.

ID	Pressure gradient [mmHg]		Surface-averaged wall shear stress [Pa]		Secondary flow degree^1^				Normalized flow displacement^1^			
					Ascending aorta		Descending aorta		Ascending aorta		Descending aorta	
	Rest	Exercise	Rest	Exercise	Rest	Exercise	Rest	Exercise	Rest	Exercise	Rest	Exercise
1	10	22	5.5	7.3	0.163	0.160	0.633	0.576	0.036	0.035	0.123	0.125
2	10	23	12.8	20.6	0.201	0.261	0.280	0.281	0.015	0.066	0.113	0.163
3	50	70	44.7	33.7	1.483	1.253	0.303	0.223	0.197	0.221	0.036	0.076
4	13	25	30.8	41.2	0.326	0.260	0.264	0.319	0.089	0.035	0.089	0.130
5	11	18	8.3	10.4	0.157	0.152	0.199	0.207	0.055	0.055	0.051	0.055
6	55	78	34.7	47.4	0.217	0.312	0.853	0.936	0.063	0.047	0.192	0.228
7	43	55	9.1	10.5	0.187	0.142	1.825	1.673	0.012	0.025	0.074	0.068
8	23	44	28.6	30.5	0.594	0.284	0.136	0.138	0.111	0.063	0.115	0.079
9	12	33	19.1	26.8	0.428	0.321	0.357	0.430	0.076	0.030	0.198	0.208
10	5	3	11.1	8.8	1.286	0.960	0.226	0.264	0.153	0.058	0.034	0.043
11	3	5	8.3	10.2	0.947	0.866	0.244	0.370	0.102	0.147	0.155	0.161
12	45	63	37.0	59.9	0.173	0.173	0.277	0.140	0.021	0.021	0.069	0.156
13	4	4	20.5	23.4	0.716	0.677	0.789	0.757	0.212	0.163	0.163	0.194
14	22	34	38.7	38.3	0.324	0.313	0.116	0.115	0.142	0.129	0.017	0.017
15	5	8	5.2	8.1	0.176	0.500	1.523	2.942	0.018	0.045	0.209	0.023
16	15	25	20.3	35.3	0.603	0.553	0.617	0.760	0.135	0.154	0.143	0.194
17	12	22	22.5	35.0	0.435	0.363	0.337	0.286	0.051	0.046	0.166	0.126
18	2	7	22.2	22.7	0.079	0.078	0.356	0.411	0.027	0.027	0.086	0.070
19	13	16	21.6	25.9	0.509	0.236	0.397	0.258	0.124	0.106	0.060	0.058
20	7	15	5.6	11.6	0.241	0.486	0.241	0.490	0.048	0.127	0.052	0.126

While the cardiac index increased for all patients during exercise, the stroke volume index was reduced during exercise for six patients, which are highlighted in Fig. 4. Those patients had lower resting heart rates than the patients who featured a stroke volume increase during exercise (61.2 vs. 89.9 bpm, p < 0.001). Also, with exception of one patient, they all were in the lower part of the cardiac index distribution (3.2 vs. 4.0, p < 0.05 at rest and 4.3 vs. 5.6, p < 0.05 during exercise). No relevant difference in the increase of the transstenotic pressure gradient was observed between those two groups (8.9 vs 11.1 mmHg, p = 0.55). The measured maximum volume flow rate in the ascending (rest 407.0 ± 87.3, stress 494.4 ± 225.5 ml/min, p < 0.001) and descending (rest 225.5 ± 76.7, stress 274.8 ± 83.8 ml/min, p = 0.002) aorta significantly increased during exercise. However, the ratio of the peak-systolic volume flow rate measured in the ascending and descending aorta did not change significantly (p = 0.287). An overview of changes in hemodynamic parameters is provided in Table 2.

## Discussion

The major finding of this study is, that MRI ergometry in combination with CFD can be used to noninvasively assess transstenotic pressure gradients, aortic flow patterns and stroke volume in patients with aortic coarctation at rest and during physical exercise. Therefore, no simulation of exercise using adrenergic drugs is necessary. This information has promising implications for clinical application and further research on the pathophysiology of aortic coarctation.

### Practical considerations

Exercise testing inside the scanner bore was tolerated well by all patients. Time taken for preparation was approximately 15 min, image acquisition took 15 min at rest and 5 min during exercise. Image quality depended on the patients’ ability to minimize movements of the upper body during exercise and could be enhanced by further fixation of the upper body to the scanner table. As 4D VEC MRI data require averaging across multiple heart cycles, images acquired during exercise were noisier compared to rest. However, in all cases image quality was sufficient to determine pressure gradients, left ventricular stroke volume and flow profiles.

The number of finite volume elements used for the simulations varied from 0.6 to 3.6 million with an average of 1.6 million elements. Using 18 out of 20 CPU cores on a local Workstation with two Xeon E5-2630 v4 CPUs, calculations took two to six hours, while the largest simulation required 6.8 Gigabyte of RAM. This duration can be reduced to under ten minutes by using high performance computing. Despite these additional expenditures in imaging and data processing time, the method can still be regarded as time-efficient compared to the expenses of a cardiac catheter examination.

### Noninvasive assessment of pressure gradients

Although current guidelines mainly focus on resting gradients in therapy planning^28^, pressure gradients may increase during exercise^5,29^, uncovering a relevant stenosis. Correspondingly, pressure gradients of several patients in this study were normal at rest but increased to levels above the thresholds for intervention during exercise. To determine pressure gradients during exercise in the heart catheter setting, adrenergic drug infusion is typically used. However, such pharmacological stress testing was shown to provoke non-physiological hemodynamic responses compared to physical exercise, with lower increases in heart rate, stroke volume and cardiac output in healthy adults^19^. In addition, Cnota et al. observed lower cardiac indices despite higher contractility, due to a lower preload during pharmacological stress^10^. Despite lower cardiac indices, gradients in patients with aortic stenosis were found to be higher during pharmacological stress, potentially due to a larger decrease of vascular resistance behind the stenosis^11^. A decrease in blood pressure proximal and distal to the stenosis was also observed in patients with aortic coarctation^5^.

### Hemodynamic response to exercise beyond pressure gradients

In this study, the average pressure gradient increased during exercise as the cardiac output increased as well. While this is no surprising finding, our results demonstrate, that assessment of the individual response to dynamic exercise is important. Whether the cardiac index increased only due to an increase of heart rate or also due to an increase in stroke volume differed between individuals. However, that the reaction of stroke volume to exercise does not allow an estimation of the pressure gradient, further emphasizes the need to individually evaluate the different parameters of hemodynamic reaction to exercise. Also, differences in the flow partitioning between head and arms and the descending aorta during rest and exercise were observed in individual cases. Therefore, those aspects, that regulate the peak-systolic flow rate passing through the aortic coarctation and thus the change in the transstenotic pressure gradient, will be discussed separately at the end of the following subsection.

Even though transstenotic pressure gradients are currently decisive in the clinical decision-making process, certain complications of aortic coarctation cannot solely be explained by them. Aortic aneurysm formation as well as intracranial vascular alterations and the premature occurrence of haemorrhagic and ischemic stroke have been reported in patients with aortic coarctation^3,30^ and were associated with altered blood flow^31^.

Increased WSS has been associated with aortic dilatation^32,33^, endothelial cell dysfunction^34,35^, development of atherosclerotic plaque^36^, as well as the formation of intracranial aneurysms^31^. WSS and secondary flow have already shown to be increased in patients with aortic coarctation^37,38^ at rest. In this study, an increase in WSS during exercise was observed. During exercise, the systole accounts for a larger relative portion of one heartbeat^39^. Therefore, cycle-averaged WSS is not only increased due to the increase of the peak-systolic magnitude of WSS, but due to the reduced diastolic time. This indicates that evaluation of WSS not only at rest but during exercise might be warranted to better understand this parameter’s effect on vascular remodelling.

Flow displacement, which was in this study measured using NFD, has been associated to the ascending aortic diameter and aortic growth in patients with bicuspid aortic valve^40,41^, a common comorbidity of aortic coarctation. However, in this study SFD and NFD remained unchanged during exercise. As it has been reported that abnormal velocity profiles are associated with previous treatment^42^, it may be due to the heterogeneity in previous treatments of this study’s cohort that only small changes in the overall cohort were found.

Additionally, the presented approach allows quantification of flow rates in the ascending and descending aorta at rest and during exercise. This allows a quantification of blood flow through the branching vessels of the aortic arch, reaching the cerebral circulation or collaterals. However, the ratio of blood flow in the ascending and descending aorta did not change during exercise on average.

Analysing aortic flow patterns, and how they change during physical exercise, may provide valuable information, as for example wall shear stresses or oscillating shear indices, regarding the role of abnormal blood flow for the development of atherosclerotic plaque or dilatation of the aorta.

Another consequence of the aortic narrowing is an increased pressure load of the left ventricle, which can on long term lead to adverse remodelling of the left ventricle^43^. Though many patients with aortic coarctation show a preserved left ventricular systolic function at rest^2,44^, the contractile response and systolic and diastolic function reserve was reported to be reduced during exercise^44^. Correspondingly, only a minimal increase in stroke volume during exercise was observed^45^.

In line with these findings, stroke volume did not significantly increase during exercise in our study. The increase in cardiac index was mainly driven by an increase in heart rate. However, the individual responses to exercise differed strongly between patients even though the same heart rate increase was targeted for every patient. The changes in cardiac index due to exercise ranged from almost none to a two-fold increase. Changes in peak-systolic flow rate varied similarly. This may be relevant, as in cases of an increase in cardiac index without a relevant change in the peak-systolic flow rate, no increase of the transstenotic pressure gradient is to be expected. Furthermore, an increase in heart rate is achieved by a decrease of the diastolic phase, whereas the systolic phase remains unchanged^39^. Thus, the relative duration of the systole per heartbeat increases, whereas the relative duration of the diastole decreases. Consequently, the left ventricle and the aorta are affected by the high-pressure condition during systole for a larger relative time.

The patient-specific differences in hemodynamic responses to exercise support the importance of individual measurements. Whether the individual differences can be attributed to different levels of ventricular dysfunction, caused by aortic coarctation, cannot be answered by this study. However, combining the presented approach with image-based methods for quantification of myocardial power might allow to identify underlying mechanisms^46^ .

### Limitations

The study design does not include a validation of the calculated pressure gradients against data from invasive catherization. However, MRI-based CFD methods for assessing pressure gradients at rest were repeatedly validated against invasive heart catheterization as a reference standard^12–15^. As this study’s aim was to demonstrate the feasibility of the combination of MRI ergometry and MRI-bases CFD, no repeated validation was included within the study design. Furthermore, the infusion of adrenergic drugs can only incompletely mimic the changes found during physical exercise. Comparing pressure gradients determined from pharmacological stress testing during catheterization to the MRI-ergometry approach was not considered to be beneficial. Furthermore, prospective assessment of catheter-measured pressure gradients is not possible due to existing risks for vascular injury, bleeding, and cardiac complications^47^. There is currently no valid standard for invasive measurement of pressure gradients during dynamic exercise. Validation against 4D flow MRI is also not possible as movement of the patients during dynamic exercise as well as the metallic stents prohibit volumetric velocity measurements. In future studies, in-vitro experiments for validation of the presented method might be warranted to quantify the accuracy of predictions. Also, 4D flow measurements at rest could be used to perform a patient-specific validation of the numerical simulation at rest, which would allow a reducing the uncertainty for the stress simulations as well.

Furthermore, this study did not include a healthy control group. Thus, the individual responses of stroke volume and cardiac index to physical exercise were not directly compared to the responses in healthy subjects. However, MRI-ergometry was already used in previous studies and was shown to induce physiological responses to exercise. Also, individual responses to exercise were compared against findings from literature, showing good agreement. As the main focus of this study was the calculation of the pressure gradients, no control group was enrolled, since no relevant pressure gradient across a non-stenosed aorta is to be expected during either rest or exercise.

Finally, approximately half of the patients investigated in this study were already treated using stents. This resulted in relatively small pressure gradients on the one hand and a more difficult segmentation of the stented region on the other hand. However, the accuracy of the patient-specific anatomy’s reconstruction is known to influence the estimation of trans-stenotic pressure gradients. Therefore, it might be warranted to use additional information available, as for example angiography data acquired during the intervention, to improve image segmentation. Nonetheless, the sample is a common representation of adolescent and young adult patients with coarctation of the aorta. Those patients require permanent monitoring and have a high risk for re-stenosis or persistent arterial hypertension. While a sample containing only patients without treatment would be favourable, this would require a large multi-centric effort as many patients are treated within the first year after birth.

## Conclusion

In this feasibility study, MRI-ergometry and image-based computational fluid dynamics were combined. We were able to demonstrate that this approach allows assessing the individual hemodynamic response to exercise under nearly physiological conditions. As pressure gradients at rest and during physical exercise can be determined noninvasively, it has the potential to serve as an alternative to pharmacological stress testing during cardiac catheterization. While the numerical method used has been validated previously, a thorough validation of the translation towards assessment of pressure gradients during dynamic exercise is required before clinical application. However, as currently no clinical standard for measurement of the transstenotic pressure gradient during dynamic exercise exists, this combined approach seems promising. In general, the method has the potential for measuring individual changes in transstenotic pressure gradients, aortic flow patterns, the left ventricular response to dynamic exercise, providing valuable information for studying the pathophysiology of aortic coarctation. Currently, those parameters can only be assessed in a very limited matter or cannot be assessed at all during dynamic exercise.

## Data Availability

The datasets generated during and/or analysed during the current study are available from the corresponding author on reasonable request.
